# Effect of different cavity designs and CAD/CAM blocks on fracture resistance of maxillary premolars with MOD cavities

**DOI:** 10.1007/s10266-025-01079-4

**Published:** 2025-03-18

**Authors:** Mohamed F. Haridy, Hend S. Ahmed, Radwa Nagy Ahmed, Shaimaa Elsayed Elhusseiny

**Affiliations:** 1 Department of Conservative Dentistry, Faculty of Dentistry, The British University, Al Shorouk City, Egypt; 2 Department of Conservative Dentistry, Faculty of Dentistry, Cairo University, Cairo, Egypt; 3 New Cairo Hospital, Ministry of Health and Population, Cairo, Egypt

**Keywords:** Lithium disilicate, CAD/CAM, Composite blocks, Overlay, Inlay, Indirect, Restoration

## Abstract

The aim of the study was to evaluate the fracture resistance of maxillary premolars restored by different CAD/CAM blocks with different MOD cavity designs. A total of 56 maxillary premolars were selected and randomly divided into 4 groups. I: intact teeth as a positive control group. Standardized MOD cavities were prepared in the remaining group specimens. II: teeth had MOD cavities but were left unrestored as a negative control group. III: MOD preparations restored with inlays with no cusp reduction. IV: MOD preparations restored with overlays with cusp reduction. Group III and IV were further subdivided into two subgroups according to material used, i.e., either lithium disilicate or composite CAD/CAM blocks. All specimens were subjected to 5000 cycles of thermocycling and then tested for fracture resistance. Failure patterns were also examined. Data were statistically analyzed using Welch one-way ANOVA followed by Games–Howell’s post hoc test. The results showed significant differences among the experimental groups (*p* < 0.001). The highest fracture resistance value was observed in positive control group, followed by overlays restored with lithium disilicate blocks. This was followed by overlays restored with composite blocks, then inlays restored by lithium disilicate blocks, inlays restored with composite blocks, while the lowest fracture resistance value was found in negative control group. Regarding failure modes, there was a significant difference between different groups (*p* < 0.001). The conclusion was that fracture resistance of maxillary premolars restored by CAD/CAM inlays and overlays are greatly affected by the cavity design and material type.

## Introduction

Restorative material selection for MOD cavities should be in such a manner that it must maintain a balance between two components: enhancing the strength of the restoration and preserving the natural tooth structure [1]. Multiple studies have reported that indirect posterior restorations exhibit better resistance to fracture compared to direct restorative techniques [2].

Recently, computer-aided design and computer-aided manufacturing (CAD–CAM) technology has become increasingly integral to the field of dentistry, enabling designing and fabrication of restorations through automated computer-assisted techniques. This growth has led to the introduction and adaptation of new materials, including ceramics, resin composites, and hybrid materials, specifically for milling restorations using CAD–CAM technology [3].

CAD/CAM ceramics are acknowledged as one of the premier materials utilized in the field of dentistry. They offer excellent esthetics coupled with sufficient strength [4]. One of the most frequently used types of ceramics is glass-based lithium disilicate. These polycrystalline materials are formed through controlled crystallization of glasses via heat treatment, resulting in crystalline phases within a glassy matrix. However, the physical characteristics of lithium disilicate glass ceramics change according to several parameters. Microstructure significantly influences flexural strength, flexural toughness, modulus of elasticity, and optical characteristics. The microstructure is governed by various parameters, including chemical structure, additives, nucleating agents, and heat treatment procedures [5]. Therefore, all newly introduced materials, due to their varying compositions, require ongoing investigation to fully understand their properties, optimize their applications, and to take the maximum advantage of their chemical durability, biocompatibility, and resistance to fracture and wear [6, 7].

Nonetheless, glass-based lithium disilicate is a stiff and fragile material [8]. Several attempts have been suggested to employ materials with an elasticity modulus comparable to that of dentin for achieving a more balanced stress distribution. Consequently, a recently introduced machinable resin composite, incorporating nanoceramic fillers and perceived as an organic combination of resin, amorphous silica and dental glass, has been introduced [9].

Composite resin CAD/CAM blocks have been introduced with excellent wear resistance and low elasticity modulus which enables its application in different designs and allowing for more conservative preparation [10]. Being polymerized under elevated temperature and pressure, CAD/CAM composite blocks exhibit enhanced biological and mechanical properties compared to conventionally light-cured composites, achieved through a significant increase in the degree of conversion [11]. However, the efficacy of machinable composite resin in minimally invasive restorations remains to be debated. Fracture resistance, evaluated with a Universal Testing Machine (UTM), is a crucial parameter that offers insights into a material or structure’s capacity to withstand fracture or failure when subjected to an applied load [12].

Therefore, the aim of the present study was to examine fracture resistance of maxillary premolar teeth restored by CAD/CAM glass-based lithium disilicate versus CAD/CAM resin-based composite materials with different designs of mesio-occlusal-distal (MOD) cavity preparations (without cuspal coverage or with cuspal coverage). The null hypothesis was that there would be no statistically significant difference in fracture resistance between two preparation types (inlay and overlay) using both resin composite and glass-based lithium disilicate CAD/CAM blocks to restore MOD cavities in maxillary premolars.

## Materials and methods

All materials’ specifications, compositions, and manufacturers are presented in Table 1.

**Table 1:** Materials’ specification, compositions, manufacturers, and LOT numbers

	Specification	Composition	Manufacturer	LOT number
1- Cerec Tessera	Lithium disilicate glass–ceramics	Two essential crystals: lithium disilicate (Li2Si2O5) and virgilite (Li0.5Al0.5Si2.5O6), which is a LAS (Lithium Aluminum Silicate) type of crystal	Dentsply Sirona, Ballantyne CorporatePl., Charlotte, NC	16011188
2- Brilliant Crios	Nano-hybrid resin composite blocks	1. Dental glass Barium glass Size < 1.0 µm 2. Amorphous silica SiO2 Size < 20 nm 3. Resin matrix cross-linked methacrylates 4. Pigments Inorganic pigments such as ferrous oxide or titanium dioxide	Coltène/Whaledent GmbH Langenau / Germany	K56598
3- Fine etch 37%	Acid etching gel	Non-dripping gel consistency, 37% phosphoric acid, blue color for visual control	SPIDENT To. Ltd, Korea	FE2121470
4- IPS Ceramic	Porcelain etchant	Hydrofluoric acid 5%	Ivoclar vivadent AG, Schaan /liechtenstein	X48466
5- Aluminum oxide	Abrasive powders	4 × 85 g AquaCare Aluminium Oxide 29 Micron Powder	Velopex, Barretts Green Rd, London NW10 7AP, UK	100119
6- Porcelain primer	Silane coupling agent	Acetone, 3-(Trimethoxysilyl)propyl-2-Methyl-2-Propenoic Acid, Acetic Acid	Bisco, Schaumburg, USA	2200003351
7- Prime&Bond universal adhesive	Universal adhesive	Bi- and multifunctional acrylate, Phosphoric acid modified acrylate resin, Initiator, Isopropanol, Water	Dentsply, Sirona,Ballantyne, CorporatePl., Charlotte, NC	2106000548
8- Calibra Ceram	Adhesive dual-cured resin cement	Dimethacrylate resins; Camphorquinone Amin co-initiator; stabilizers; glass fillers; silica fillers; phosphate monomers; flouride	Dentsply Sirona, Ballantyne CorporatePl., Charlotte, NC	00100279

### Sample size calculation

A power analysis was conducted to ensure sufficient power for testing the null hypothesis that there is no significant difference in fracture resistance between two preparation types (inlay and overlay) using both composite and lithium disilicate CAD/CAM blocks. With an alpha (α) and beta (β) level of 0.05 (i.e., power = 0.95%) and an effect size (f) of 0.575, derived from a previous study [13], the minimum required sample size was determined to be 56. The sample size calculation was applied using G*Power version 3.1.9.7 [14].

### Teeth selection

This study was carried out following approval from the ethical committee of the Faculty of Dentistry at the British University in Egypt. The approval number was (21–024). A total of 56 freshly extracted sound human first maxillary premolars were selected for this study.

The teeth were selected based on the following inclusion criteria:

- No evidence of caries
- Free from any cracks or fractures in the crown
- They were extracted for either orthodontic or periodontal treatment.
- Patients age group ranged from 15–30 years and was medically free.
- The teeth were selected to have approximate similarity in anatomic crowns regarding size, shape, and length.

For standardization purposes, a digital caliper (Bacolis Digital Clipper (Stainless Haredned, Generic) was used to measure the buccolingual and mesiodistal width of the teeth. Teeth meeting the following criteria were selected for the study: buccolingual dimensions of 9 ± 0.5mm, mesiodistal width of 7 ± 0.5mm, and occluso-cervical distance of 9 ± 0.5mm, with a 0.5 mm error rate [15].

Immediately after extraction, teeth were cleaned and disinfected and were examined using 3.5 X magnification (Univet loupes, Italy) to ensure the absence of any caries, visible cracks, or hypoplastic defects, and teeth with any defects or cracks were excluded from the study.

### Grouping of specimens

**Fig. 1:**
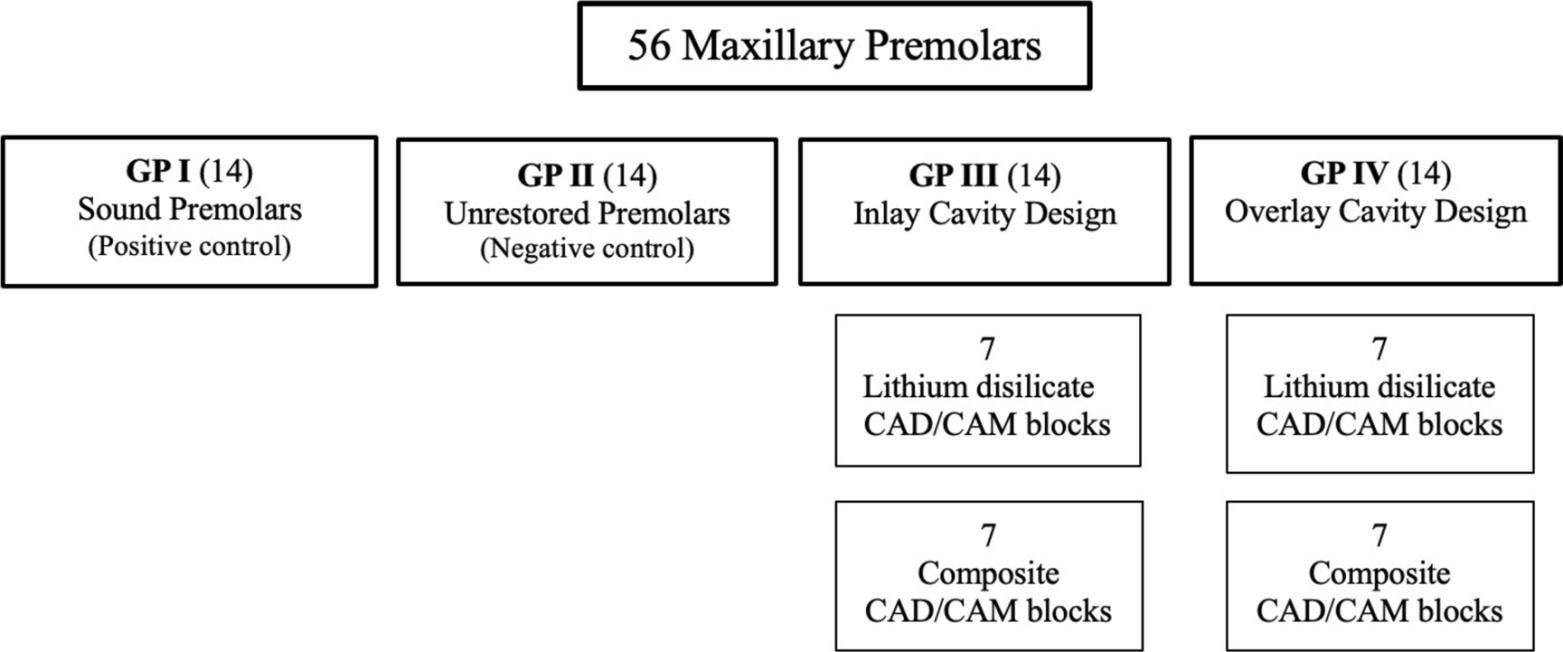
Schematic diagram for grouping of specimens

Teeth were randomly categorized into 4 main groups (14 each) based on cavity design. Gr I: teeth were left intact to act as a positive control group. Gr II: teeth were prepared with MOD cavities and then left without restoration to act as a negative control group. Gr III: teeth were prepared with MOD inlay cavity preparation. Gr IV: teeth were prepared with MOD overlay cavity preparation with both palatal and buccal cusps reduction. Both groups III and IV were further subdivided into two subgroups (seven each) according to the CAD/CAM restorative blocks used to be either restored with glass-based lithium disilicate (Cerec Tessera) or composite CAD/CAM blocks (Brilliant Crios). A schematic diagram for grouping of specimens is shown in Fig. 1.

### Preparation of specimens and cavity preparation

#### Teeth mounting and periodontal ligament simulation:

**Fig. 2:**
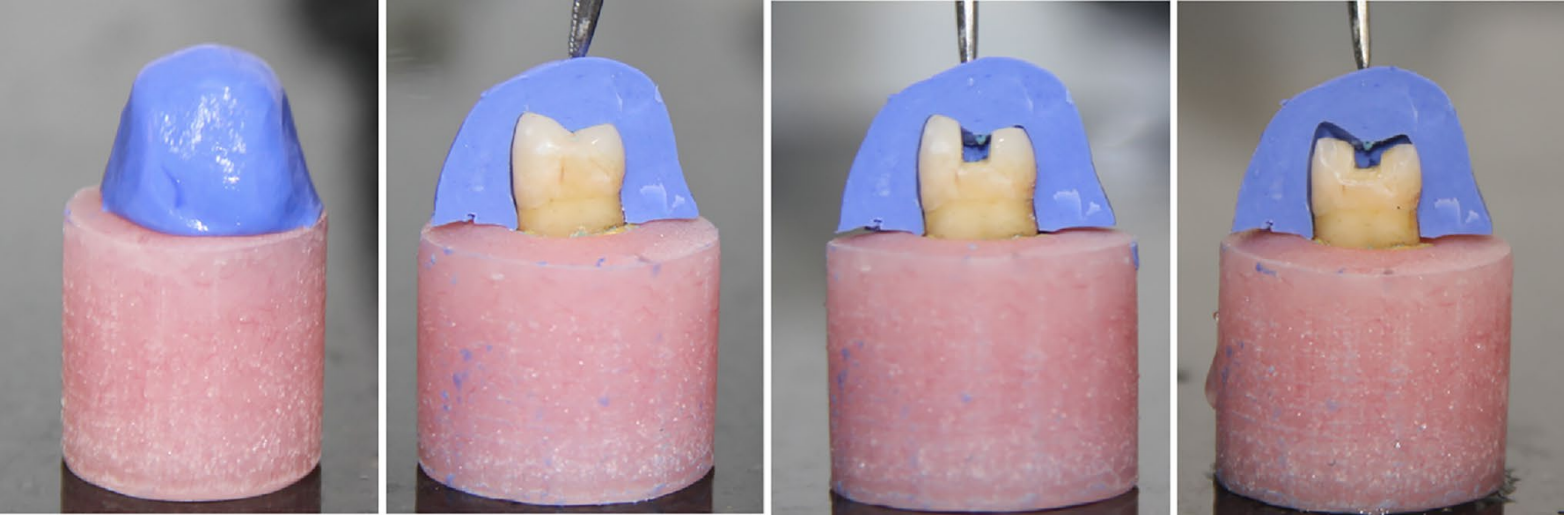
Occlusal indices fabricated from rubber base used for checking the occlusal anatomy

The roots of all teeth were dipped into melted wax (Cavex Holland, The Netherlands) to produce a 0.2 to 0.3 mm layer [16], and then embedded in a cylindrical ring (3 × 2 cm), filled with auto-polymerizing acrylic resin (Acrystone Dental Factory, England), up to 2 mm below the cement–enamel junction (C.E.J) [17]. This was done using a specially designed centralization guide device (Bredent, Milling Unit BF-2, Germany), This device was used during the mounting of each specimen to ensure that the teeth were mounted in such a way that their long axis was perpendicular to the base of the cylinder. The wax spacer was removed from the root surface using a wax knife and hot water. The acrylic resin space (alveolus) was filled with polyether impression material (Elite HD + , Zhermack S.p.A., Rovigo Italy), and allowed to be set. Heavy body silicone (Hydrorise putty- fast set, Zhermack SpA, Italy) was employed to create indices for the occlusal anatomy of the teeth to maintain standardized preparation of all specimens. Occlusal indices were fabricated from rubber base and cut into two halves mesial and distal, serving as a guide for checking occlusal anatomy in both inlay and overlay restorations (Fig. 2). This approach was used to ensure uniformity and standardization in the process [18].

#### Cavity preparation

**Fig. 3:**
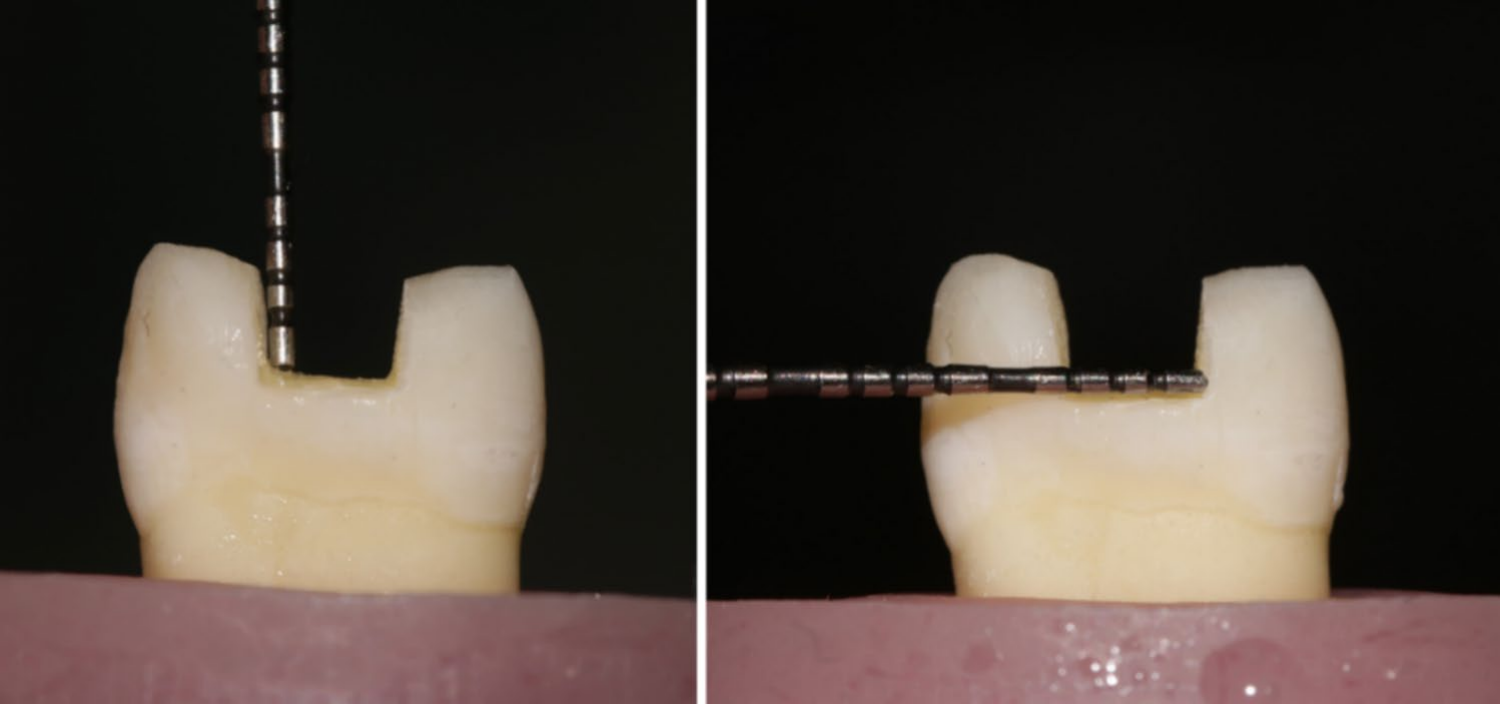
Measuring cavity depth and width of overlay design using periodontal probe

**Fig. 4:**
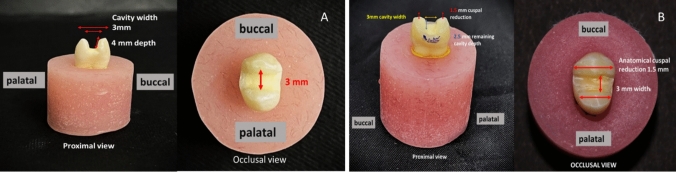
Groups II and III: **A** showing inlay cavity preparation design, **B** showing overlay cavity preparation design

For groups II, III, and IV, standardized MOD cavity preparations were prepared under magnification using 3.5 X magnification (Univet loupes, Italy) with rounded end fissure tungsten carbide burs and a high-speed handpiece under water cooling. Then blue-coded tapered abrasive with a rounded end were used to make a 4-degree taper for cavities walls [19]. The enamel margins of the MOD cavities preparations were finished using yellow-coded tapered finishing diamond abrasive. All burs and abrasives were replaced every five preparations. All specimens were prepared to have the following dimensions: occlusal width was standardized to be 3 mm. The pulpal floor depth was standardized to be 4 mm from the cusp tip as a reference point. Cavities were prepared to be rounded, diverging buccal and lingual walls of 4-degree taper, and cavo-surface angles were approximately 90 degrees. Regarding group IV, both buccal and palatal cusps were reduced evenly by 1.5 mm from the cusp tip and parallel to the occlusal plane, using a blue-coded tapered diamond stone (Fig. 3). The overlay preparation was finished using yellow-coded tapered finishing diamond stone. All preparations were done by the same operator under magnification for maximum standardization (Fig. 4).

### Fabrication of restorations from CAD/CAM blocks

**Fig. 5:**
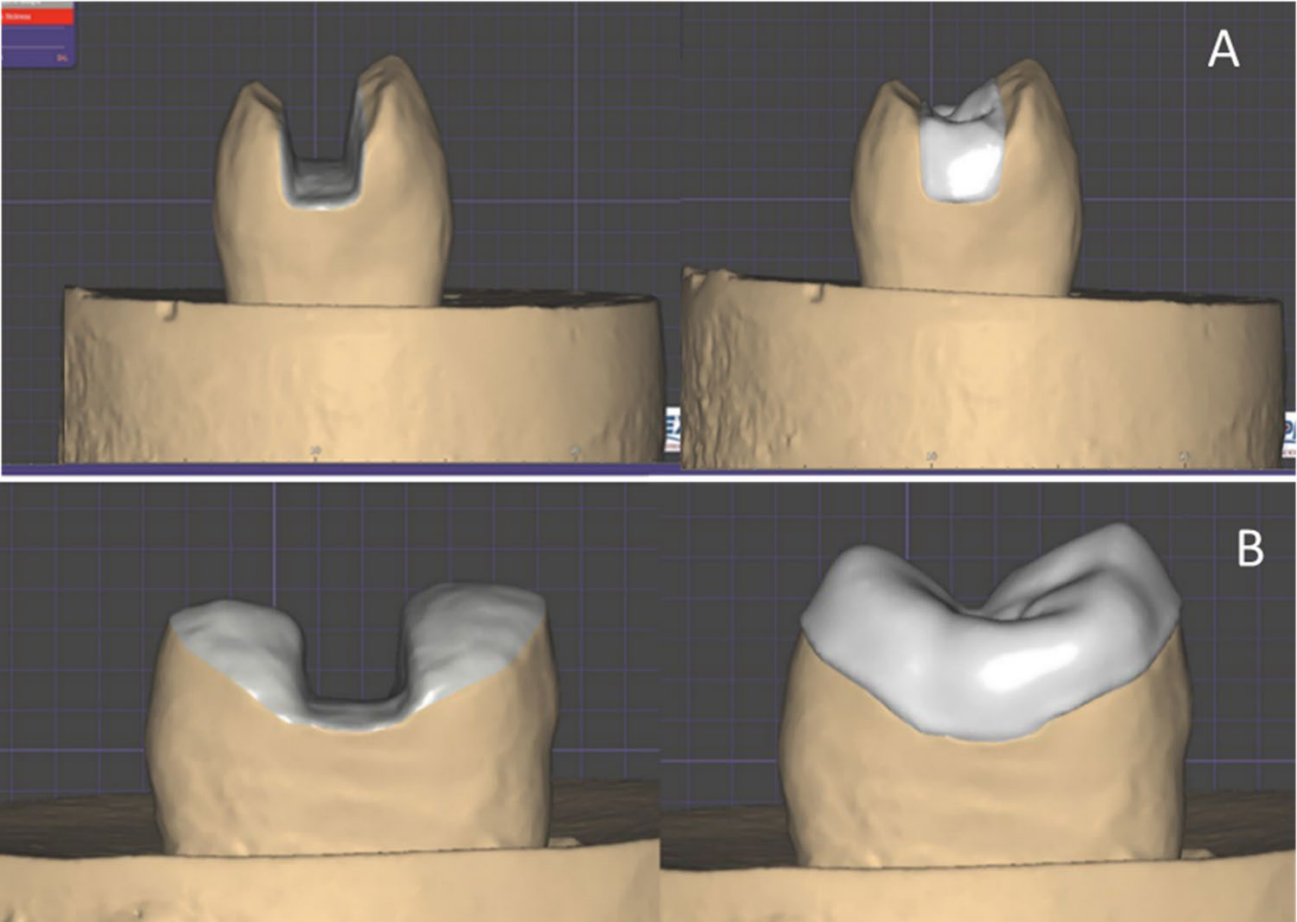
Groups II and III: **A** designing inlay samples using Exocad software, **B** designing overlay samples using Exocad software

All specimens’ preparations were scanned with Omnicam intraoral camera of the CEREC system scanner (CEREC SW5, Dentsply Sirona, York, Pennsylvania, USA) to obtain optical impressions. The scanning time of each specimen was standardized by the operator to be between 25–35 s. The restorations were designed using the software program ExoCad (exocad GmbH, Darmstadt, Germany). The cement layer of 90 μm thickness was adjusted in the software program. After successfully designing the restoration, the margins, the restoration contours, and uniformity were checked (Fig. 5). Cerec Tessera and Brilliant Crios CAD/CAM blocks were inserted in the spindle of the milling chamber and milled using milling machine (Imes-Icore 150 Ipro, Im Leibolzgraben, Germany). After milling, ceramic specimens were fired for crystallization and received a glaze layer to achieve its highest strength [20], while all composite specimens were polished according to the manufacturer’s recommendations using a Vita Enamic polishing kit [21].

### Pretreatment of CAD/CAM restorations

**Fig. 6:**
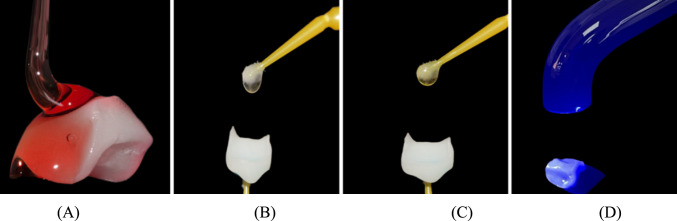
**A** Internal surfaces etching using 9.5% hydrofluoric acid (Bisco porcelain etch), **B** silane coupling agent, **C** Universal Adhesive Prime&Bond, **D** light curing using light-emitting diode (LED)

For ceramic restorations, Cerec Tessera (Dentsply Sirona, Charlotte, NC) were pretreated according to manufacturer’s instructions as follows: the fitting surface was etched with IPS ceramic porcelain etchant (HF acid 5%) for 30 s, rinsed with water for 20 s, then air dried for 10 s. Bis-Silane™ Bisco silane coupling agent was applied, left for 2 min, and then air dried. A thin layer of Prime&Bond Universal adhesive was then applied, left for 20 s, air thinned for 5 s, and light cured for 20 s (Fig. 6).

**Fig. 7:**
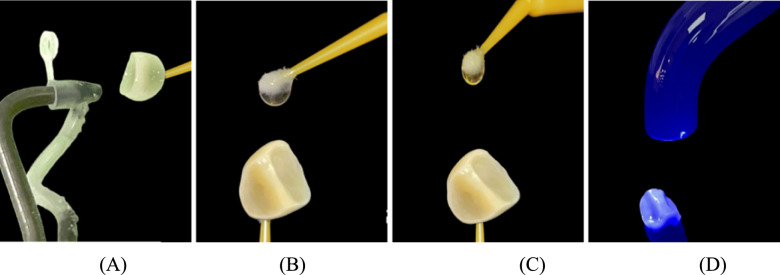
**A** Sandblasting using Aquacare dental air abrasion, **B** silane coupling agent, **C** Universal Adhesive Prime&Bond, **D** light curing using light-emitting diode (LED)

Regarding the composite restorations, Brilliant Crios (Coltène) were pretreated according to the manufacturer’s instructions as follows: the fitting surface was sandblasted for 20 s with 29 µm AquaAbrasion™ aluminum oxide at 1.5 bar. The sandblasted surfaces were then cleaned with an ultrasonic cleaner, rinsed for 20 s, air dried for 10 s, and dried. A thin layer of Prime&Bond Universal adhesive was then applied, rubbed in for 20 s, air thinned for 10 s, and light cured for 20 s. (Fig. 7).

### Pretreatment of prepared cavity before cementation

**Fig. 8:**
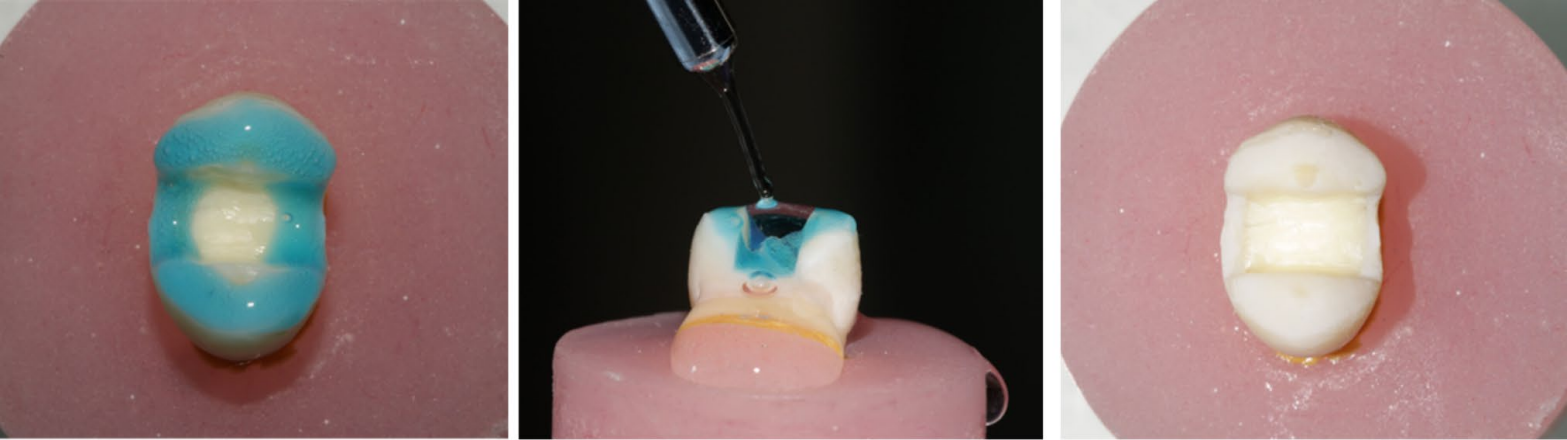
Selective etching technique (enamel only) and rinsing after 15 s

**Fig. 9:**
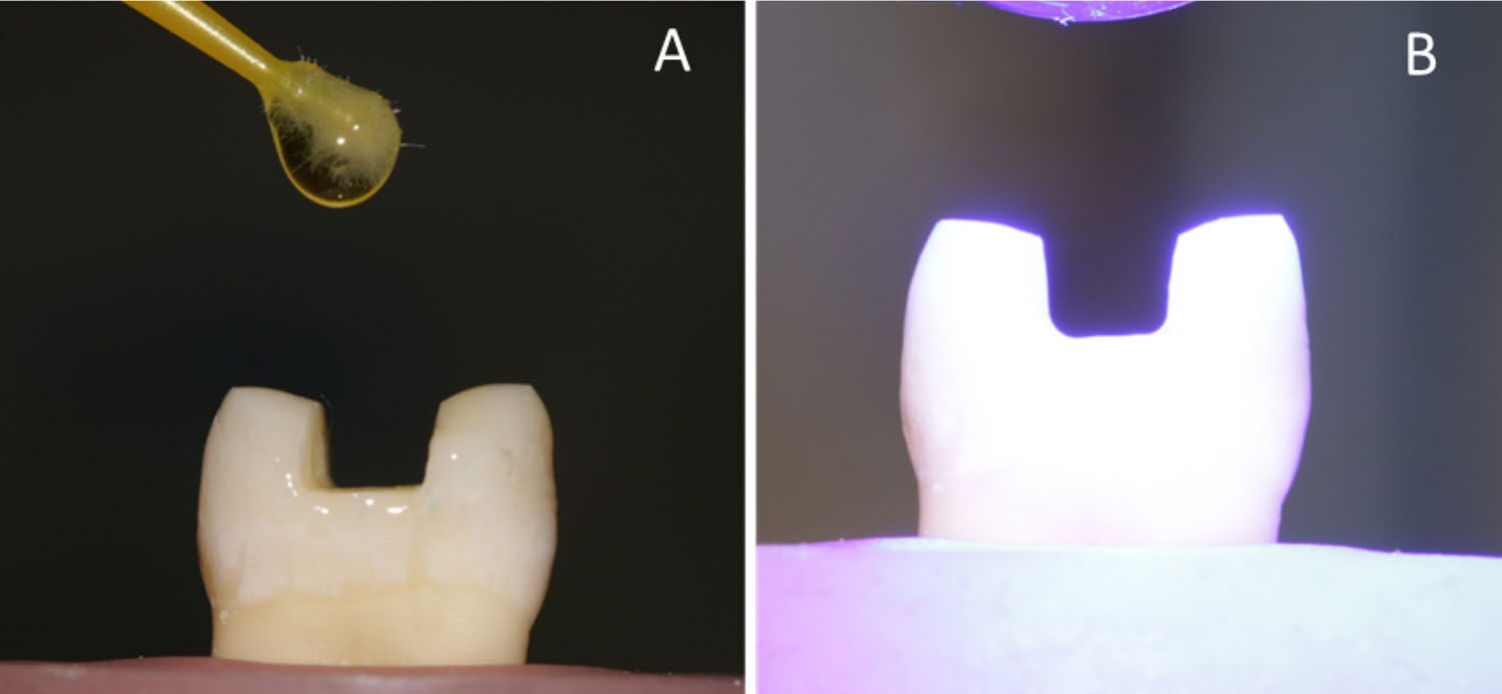
**A** A thin coat of the Universal Adhesive Prime&Bond on enamel and dentin, **B** adhesive cured using a light-emitting diode unit (LED)

A universal adhesive (Prime&Bond Universal TM, Dentsply Sirona, Japan) was used with selective etch approach. All prepared teeth in both groups III and IV were subjected to selective enamel etching using 37% for 15 s and then was rinsed with the triple air-way syringe for 15 s followed by gentle air drying for 10 s (Fig. 8). The universal adhesive was actively applied to enamel and dentin surfaces and rubbed for 20 s, air thinned for 10 s until the solvent evaporated completely, and finally was cured for 20 s (Fig. 9).

### Cementation procedure

**Fig. 10:**
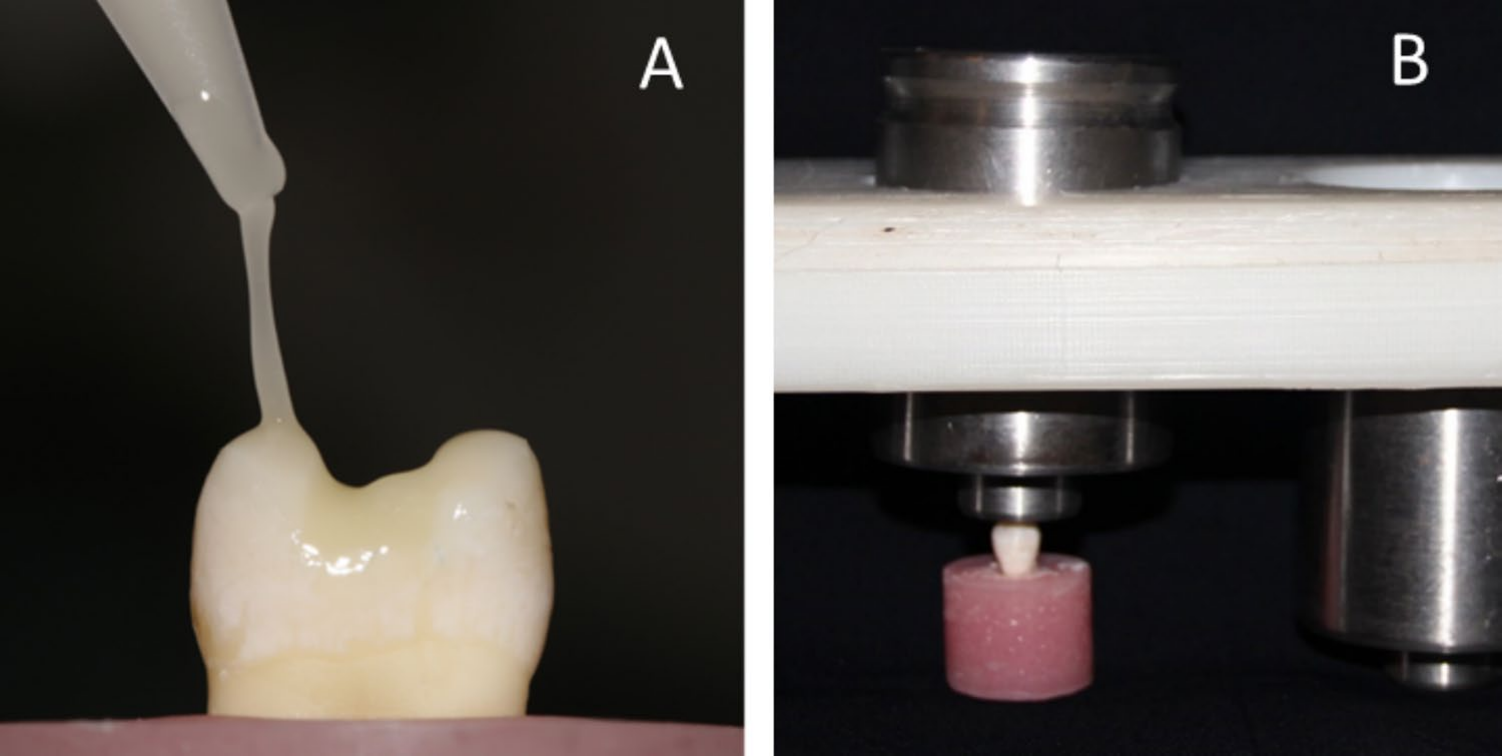
**A** A layer of Calibra Ceram resin cement, **B** custom-made static load device (1 kg)

A thin layer of Calibra Ceram adhesive resin cement (Dentsply Sirona, Charlotte, NC) was applied to the cavity (Fig. 10 A), the restoration was then fully seated with gentle finger pressure to flow excess material. A custom-made static load device (1 kg force) was then used for 2 min [22] (Fig. 10 B). The excess cement was eliminated immediately using a micro-brush. Photopolymerization was performed initially for 3 s (tack curing) followed by careful removal of the cement excess, then all margins were covered with glycerin gel (to act as oxyguard) and the curing was repeated for 20 s. In addition, each surface was light cured for 30 s to ensure maximum curing.

### Storage of specimens and thermocycling

All specimens were kept in distilled water at room temperature for a period of only 24 h before undergoing thermocycling and the fracture resistance test. After 24 h, all the specimens were subjected to 5000 cycles of thermocycling (SD Mechatronic Thermocycler Germany) at 5 ± 2 °C/55 ± 2 °C which was equivalent to approximately 6 months of clinical performance [23], and the dwell time for each cycle was 10 s.

### Fracture resistance testing and failure mode assessment

**Fig. 11:**
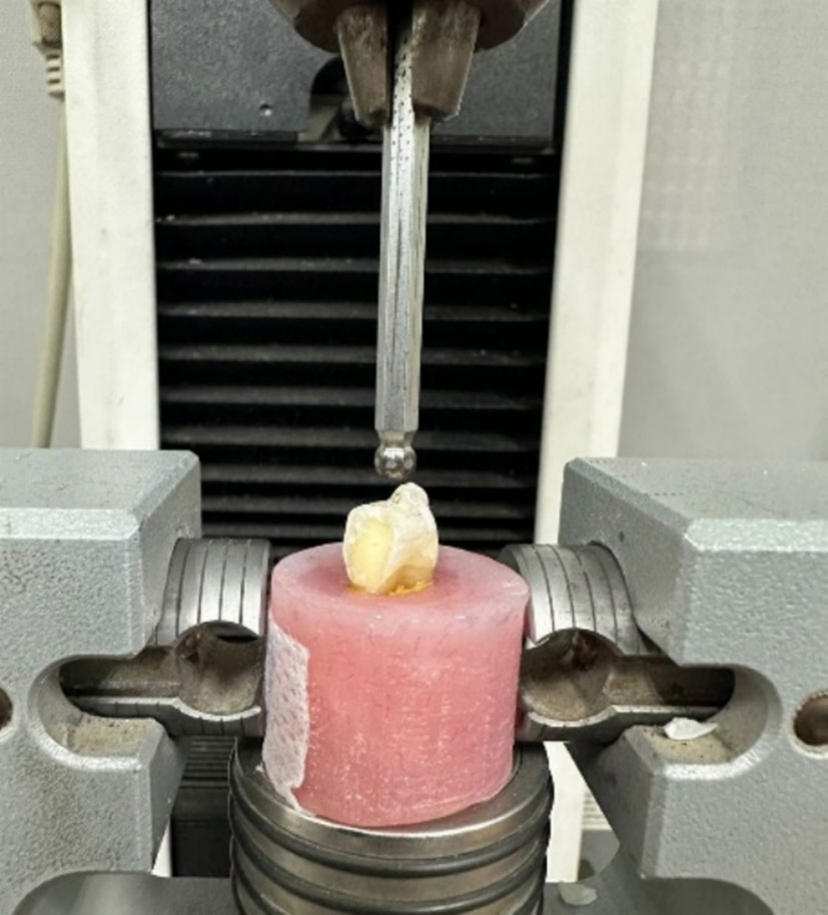
Universal Testing Machine

**Fig. 12:**
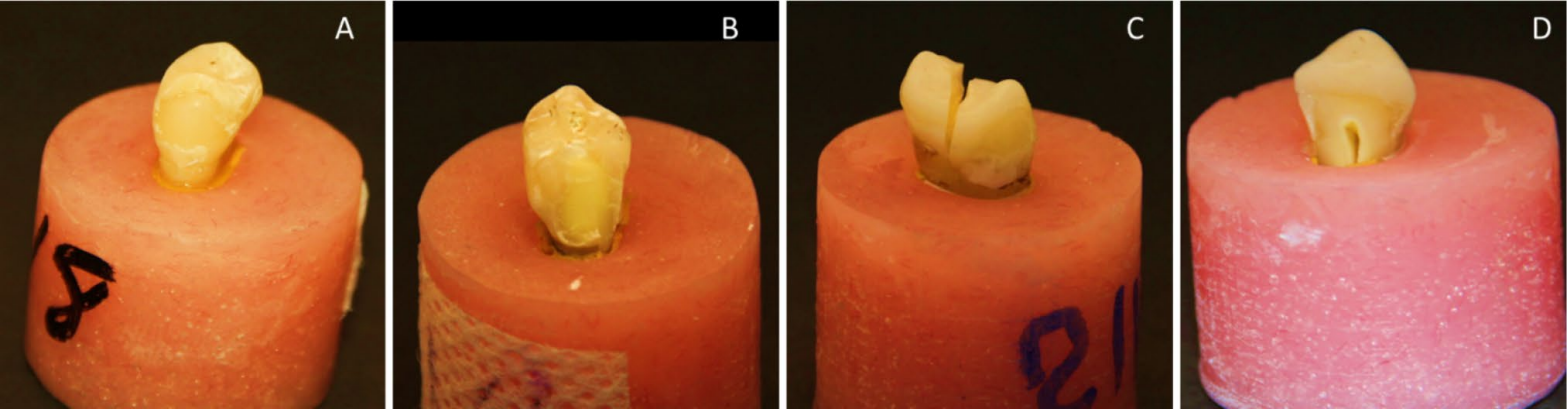
Types of failure: **A Type I:** isolated damage of the restoration, **B Type II:** fracture in a small tooth portion, **C** Type III: fracture in half of tooth above CEJ, **D Type IV:** fracture below CEJ

The fracture resistance of the specimens was assessed using a Universal Testing Machine (Instron, Model 3345, England) (Fig. 11). The tooth blocks were securely fixed in the jig of the Universal Testing Machine so that the angle between the upper rod and the long axis of the tooth was zero. Specimens were loaded axially on their occlusal surface at a crosshead speed of 1.0 mm/min. A plunger with a steel ball (4-mm diameter) was utilized to apply compressive force, making tripod contact with the cusps. The initial load was set at 20N, and the load gradually increased until a fracture occurred. Following the fracture resistance test, the fractured specimens were examined for their mode of fracture using a stereomicroscope (Olympus, Tokyo, Japan). They were then categorized into the following groups based on pattern of failure (Fig. 12). Type I: failure of the restoration only, Type II: failure in a small portion of tooth, Type III: failure in half of tooth and above CEJ, and Type IV: failure below CEJ.

### Statistical analysis

Categorical data were expressed as frequencies and percentages and analyzed using the Chi-square test, followed by pairwise comparisons with multiple z-tests and Bonferroni correction. Numerical data were represented as means and standard deviations (SD). Normality was evaluated using the Shapiro–Wilk test, which confirmed that the data were normally distributed. These data were then analyzed using two-way ANOVA, with simple main effects comparisons and *p *value adjustments using Bonferroni correction. Intergroup comparisons were performed using one-way ANOVA, followed by Tukey’s post hoc test. A significance level of *p* < 0.05 was applied to all tests. Statistical analysis was conducted using R statistical software version 4.3.1 for Windows [24].

## Results

### Fracture resistance

The fracture resistance showed a significant difference among different groups (*p* < 0.001). The highest value was observed in the positive control group, followed by lithium disilicate glass–ceramics overlay, then resin composite overlay, lithium disilicate inlay, and resin composite inlay, while the lowest value was observed in negative control group. Post hoc pairwise comparisons resulted the positive control group and lithium disilicate overlay to have a significantly higher value than other groups (*p* < 0.001). Furthermore, the composite overlay demonstrated a significantly higher value compared to both the composite inlay and the negative control group (*p* < 0.001) (Table 2).

**Table 2:** Intergroup comparisons and descriptive statistics for the fracture resistance (*N*)

Group	Mean	95% Confidence interval		SD	Min	Max
		Lower	Upper			
Lithium disilicate inlay	873.69^BC^	800.51	946.86	105.60	738.43	1018.47
Composite inlay	771.38^C^	713.37	829.39	83.71	643.88	839.08
Lithium disilicate overlay	1101.83^A^	1047.35	1156.32	78.63	991.41	1194.52
Composite overlay	973.53^B^	898.62	1048.44	108.10	801.98	1068.55
Negative control	391.70^D^	362.02	421.37	42.82	329.84	448.55
Positive control	1126.89^A^	1080.61	1173.18	66.79	1064.66	1211.66

Different superscript letters indicate a statistically significant difference within the same vertical column and horizontal row

### Fracture resistance gain %

Fracture resistance gain % was obtained by dividing the fracture resistance of the restored teeth in different groups by the positive control. There was statistically significant difference between the restorative materials and techniques (*p* < 0.001), the highest fracture resistance gain was for lithium disilicate overlay, while the least gain was for composite inlay (Table 3).

**Table 3:** Intergroup comparisons for percentage of fracture resistance gain (%)

Percentage of fracture resistance gain (*n* %)				
Fracture resistance gain (%)	Lithium disilicate overlay	Composite overlay	Lithium disilicate inlay	Composite inlay
Mean	98.00%^a^	86.44%^ab^	77.95%^bc^	68.83%^c^
SD	15.22%	6.29%	7.17%	12.54%

Different superscript letters indicate a statistically significant difference within the same vertical column and horizontal row

### Mode of failure

There was a significant difference between different groups, with lithium disilicate overlays and composite overlays having significantly higher percentage of type (I) failures than lithium disilicate inlay, positive and negative control groups, and with negative control group having significantly higher percentage of type (IV) failures than composite inlay, lithium disilicate overlay, composite overlay, and positive control (*p* < 0.001) (Table 4).

**Table 4:** Intergroup comparison, frequencies, and percentages values for mode of failure

Failure mode	*n* (%)						*p* value
	Lithium disilicate inlay	Composite inlay	Lithium disilicate overlay	Composite overlay	Negative control	Positive control	
Type I	0 (0.00%)^B^	1 (14.29%)^AB^	5 (71.43%)^A^	5 (71.43%)^A^	0 (0.00%)^B^	0 (0.00%)^B^	< 0.001*
Type II	1 (14.29%)^A^	1 (14.29%)^A^	2 (28.57%)^A^	1 (14.29%)^A^	0 (0.00%)^A^	5 (35.71%)^A^	
Type III	2 (28.57%)^A^	4 (57.14%)^A^	0 (0.00%)^A^	1 (14.29%)^A^	2 (14.29%)^A^	6 (42.86%)^A^	
Type IV	4 (57.14%)^AB^	1 (14.29%)^A^	0 (0.00%)^A^	0 (0.00%)^A^	12 (85.71%)^B^	3 (21.43%)^A^	

Different superscript letters indicate a statistically significant difference within the same vertical column and horizontal row

*Indicates significant differences

## Discussion

Indirect bonded restorations have been the clinical treatment of choice, especially in situations where placing direct restorations is challenging such as cases involving cusp fracture or extensive defects [25]. Furthermore, indirect restorations such as overlays, onlays, and inlays are considered a more conservative approach compared to full coverage restorations [26]. Indirect restorations are often preferred when achieving optimal form and esthetics is crucial. In addition, they are considered ideal for predictable full-mouth rehabilitation [27].

Maxillary premolar teeth were chosen for this investigation due to their high susceptibility to fractures compared to molars and mandibular premolars. Their susceptibility to fracture is attributed to their anatomical features, such as cuspal inclination and their unfavorable crown-to-root ratio contributes to their fragility, making them more prone to fractures under occlusal forces [28]. In addition, the position of maxillary premolars in the dental arch makes them subjected to shear and compressive forces, with occlusal forces tending to separate the buccal and lingual cusps [29].

This in vitro study aimed to assess the fracture resistance of human maxillary premolars restored with two different CAD/CAM restorative materials (lithium disilicate-based glass ceramics and resin composite blocks) and compare two types of cavity preparations (inlay and overlay preparations). In vitro research’s main goal is to mimic controlled, prospective, and longitudinal clinical scenarios in which restorations are implanted under ideal circumstances. The null hypotheses were tested that all materials show comparable fracture resistance values and different conformations of the cavity preparation would not influence fracture resistance of the restored teeth.

The findings of this study indicated that the fracture resistance and durability of restorations in MOD cavities of upper premolars are significantly influenced by both cavity design and the type of material used for restoring MOD cavities. Therefore, the *null hypothesis* tested was rejected. Overlay design restored with lithium disilicate glass ceramics had the highest fracture resistance in comparison to other designs and restorative materials.

In the present study, *overlay designs* showed higher fracture resistance than *inlay designs*. Several previous studies align with these findings [30–33]. Overlay design was reported to show more favorable distribution of occlusal forces and transferring them perpendicularly to the occlusal surface [34, 35]. Furthermore, mimicking the natural tooth anatomy by applying cuspal reduction with anatomical design, this design minimizes any potential interference with the bite, ensuring proper stress distribution across the tooth, enhancing quality of adhesion by optimizing the cutting of enamel prisms, expanding the available enamel surface, and reducing dentin exposure to a minimum [36]. In addition, the involvement of both cusps could result in an equal distribution of stresses within the cavity. Multiple previous studies suggested that covering non-functional cusps may reduce the risk of enamel cracks and potential tooth fractures in adhesively bonded restorations [37]. On the contrary, the results of this study disagree with the findings of those three other investigations [38–40] which stated that cusp coverage may decrease the fracture resistance of teeth. This could be explained by the varieties in the dimensions of cavity design in the mentioned studies and variations in the cuspal reduction design of overlay.

Regarding type restorative materials, inlay and overlay designs restored with Cerec Tessera showed significantly higher values than inlay and overlay designs restored with Brilliant Crios. These findings agree with many other studies [41–43]. The distinction in fracture resistance esteems among the two CAD/CAM materials tested might be due to their different chemical compositions and microstructures. It was reported that lithium disilicate glass–ceramics have the ability to restore tooth structure rigidity more effectively than indirect composite restorations. In addition, lithium disilicate with high elastic modulus tend to concentrate more stresses inside the ceramics, while the indirect resin composite restorations with low elastic modulus tends to transmit more stress to the tooth structure which may put teeth at a greater risk of fracture [44], Also this was confirmed statistically by measuring the percentage of fracture resistance gained. Lithium disilicate overlays restore 98% of fracture resistance, while composite overlays restore 86.4%.

Controversy, other studies [45, 46] have yielded different findings that CAD\CAM resin composite overlay showed higher fracture resistance than CAD\CAM lithium disilicate-glass overlay. This difference may be attributed to the fact that these studies examined indirect overlay restoration with only 1 mm thickness. CAD\CAM resin–composite blocks have shown more load absorbent in thin thickness than brittle ceramics. There was also other discrepancy in findings with other studies [47, 48] in as far as the fracture resistance of composite inlays was higher than that of ceramics overlays and that could be attributed to various factors, including the type of ceramic material used, which was feldspathic ceramics, and differences in the type of composite and resin cement employed, as compared to the current study.

In the current study, fracture mode analysis revealed more favorable outcomes for overlay designs restored with both lithium disilicate and resin composite blocks. The majority of specimens had type I failure, and the difference between the two tested restorative CAD\CAM materials was not statistically significant. This comes with the agreement of several studies [49–51] which concluded that both lithium disilicate and resin composite blocks could protect tooth structure and prevent transmission of stresses to the underlying tooth structure. In addition, findings of this study showed a significant tendency to cusp separation in teeth restored with inlay designs which revealed fracture failure type III. and IV. This comes in agreement with several studies [52] which suggest that the increased risk of fracture in inlay cavities may be attributed to the wedge effect, as it generates additional horizontal stresses on the cavity walls, concentrate stresses, leading to the formation and propagation of cracks, resulting in fracture and structural failure.

Regarding groups with CAD/CAM resin composite inlay design, more favorable outcomes were observed, with Type III fractures above the CEJ. In contrast, inlays restored using lithium disilicate-glass exhibited less favorable fracture patterns with Type IV involving the root and rendering the tooth unrestorable. These variations may be attributed to the differences in their elasticity modulus. Resin composite materials, with an elasticity modulus similar to dentin, tend to bend under load, distributing stresses evenly and offering stress-absorbing properties [9]. Conversely, the rigidity of lithium disilicate-glass ceramics can lead to stress concentrations at critical areas, potentially causing catastrophic failures [53, 54].

Clinically, maxillary premolars with MOD cavity are highly prone to fracture due to enamel discontinuity by losing the two marginal ridges that make them more susceptible to wedging, cusp deflection and breakage action by the restoration during mastication. This could be attributed to the combination of their unfavorable structure (in terms of crown volume and crown/root proportion) and the complex lateral stresses enhanced by their position in the dental arch. This explains why premolars with MOD cavity design were used in this study to replicate the most catastrophic situation for posterior teeth [55]. Accordingly, the ideal restorative approach of such cases should establish a balance between maximizing both the functionality and esthetic of the restored tooth while minimizing the risk of subsequent tooth fracture [56]. Bondable restorations, typically dental ceramics and resin-based materials along with durable and stable dental adhesive system and luting resins are very crucial for the successful restorations of mutilated posterior teeth [57–59]. The choice of indirect restorations over the direct ones is highly advocated to restore the lost fracture resistance due to loss of tooth structures. CAD–CAM blocks either of ceramics or composite could be used [58, 59]. Recommendations of using lithium disilicate with the prepared designs was in accordance with Comba et al. in 2022 where they stated that lithium disilicate showed high fracture resistance when physiological and increased loading were applied which was also in agreement to previous studies [60–62]. Finally, it was observed that the maximum biting force for a single posterior tooth was around 725 N [63]. In this investigation, the fracture loads applied surpassed the maximum biting forces, potentially simulating situations of overloading such as bruxism or traumatic occlusion. The result of this study showed that both tested restorative materials bonded to either inlay or overlay cavity design survived this force, hence both indirect restorations succeeded in reinforcing a weakened MOD cavity in premolar with superior performance of overlay designs restored with lithium disilicate CAD/CAM restorations.

This study was limited to static compressive strength testing. It is recognized that static loading does not fully stimulate the long-term effects of occlusal forces on the restoration-tooth system, nor the forces exerted by patients with occlusal wear. While the static load value may represent the maximum strength of the tested restorations, environmental factors and cyclic loading are likely to reduce this strength over time [64]. Therefore, further research on dynamic strength testing is recommended.

## Conclusion

Considering the limitations of the present study, the following can be concluded: the fracture resistance and failure patterns of maxillary premolars restored with CAD/CAM inlays and overlays are significantly influenced by both cavity design and material type. While MOD inlays offer notable reinforcement of tooth structure, occlusal overlays provide superior reinforcement. Among the materials studied, lithium disilicate ceramic overlays proved to be the most effective in restoring MOD cavities in maxillary premolars.

## Data Availability

Data are available upon reasonable request from the corresponding author.
